# Multitarget Multiscale Simulation for Pharmacological Treatment of Dystonia in Motor Cortex

**DOI:** 10.3389/fphar.2016.00157

**Published:** 2016-06-14

**Authors:** Samuel A. Neymotin, Salvador Dura-Bernal, Peter Lakatos, Terence D. Sanger, William W. Lytton

**Affiliations:** ^1^Department Physiology and Pharmacology, SUNY Downstate Medical Center, State University of New YorkBrooklyn, NY, USA; ^2^Department Neuroscience, Yale University School of MedicineNew Haven, CT, USA; ^3^Nathan S. Kline Institute for Psychiatric ResearchOrangeburg, NY, USA; ^4^Department Biomedical Engineering, University of Southern CaliforniaLos Angeles, CA, USA; ^5^Division Neurology, Child Neurology and Movement Disorders, Children's Hospital Los AngelesLos Angeles, CA, USA; ^6^Department Neurology, SUNY Downstate Medical CenterBrooklyn, NY, USA; ^7^Department Neurology, Kings County Hospital CenterBrooklyn, NY, USA; ^8^The Robert F. Furchgott Center for Neural and Behavioral ScienceBrooklyn, NY, US

**Keywords:** dystonia, multiscale modeling, computer simulation, motor cortex, beta oscillations, corticospinal neurons, multitarget pharmacology, support vector machines

## Abstract

A large number of physiomic pathologies can produce hyperexcitability in cortex. Depending on severity, cortical hyperexcitability may manifest clinically as a hyperkinetic movement disorder or as epilpesy. We focus here on dystonia, a movement disorder that produces involuntary muscle contractions and involves pathology in multiple brain areas including basal ganglia, thalamus, cerebellum, and sensory and motor cortices. Most research in dystonia has focused on basal ganglia, while much pharmacological treatment is provided directly at muscles to prevent contraction. Motor cortex is another potential target for therapy that exhibits pathological dynamics in dystonia, including heightened activity and altered beta oscillations. We developed a multiscale model of primary motor cortex, ranging from molecular, up to cellular, and network levels, containing 1715 compartmental model neurons with multiple ion channels and intracellular molecular dynamics. We wired the model based on electrophysiological data obtained from mouse motor cortex circuit mapping experiments. We used the model to reproduce patterns of heightened activity seen in dystonia by applying independent random variations in parameters to identify pathological parameter sets. These models demonstrated degeneracy, meaning that there were many ways of obtaining the pathological syndrome. There was no single parameter alteration which would consistently distinguish pathological from physiological dynamics. At higher dimensions in parameter space, we were able to use support vector machines to distinguish the two patterns in different regions of space and thereby trace multitarget routes from dystonic to physiological dynamics. These results suggest the use of *in silico* models for discovery of multitarget drug cocktails.

## 1. Introduction

A large number of physiomic pathologies can produce hyperexcitability in cortex. In motor cortex, this hyperexcitability will manifest as alterations in movement and muscle tone. At the most extreme, hyperexcitability leads to a seizure with uncontrolled movement, as seen in epilepsia partialis continuans. Lesser hyperexcitability produces a variety of hyperactive movement disorders, including tics, chorea, tremor, etc, whose pathophysiology is not restricted to cortex, but involves multiple brain areas including basal ganglia, thalamus, cerebellum, and others. We focus here on dystonia, a movement disorder that produces prolonged involuntary muscle contractions (Neychev et al., 2008; Crowell et al., 2012).

The large variety of dystonias of different etiologies may present with involvement of one or several parts of the body. Pediatric causes of dystonia include cerebral palsy and are generally distinct from adult-onset cases. Common adult dystonias are torticollis, causing involuntary head turning, and movement-overuse dystonias such as writers cramp. Despite these differences, dystonias in different patient populations are primarily treated with the same therapies. While most research in dystonia has focused on basal ganglia, much pharmacological treatment is provided directly at muscles. Similarly, we propose that treatment could be targeted elsewhere in the motor pathway, here focusing on motor cortex as a potential target for therapy.

As with many other movement disorders, the dystonias generally lack a reliable biomarker and are diagnosed by semiology, the assessment of signs and symptoms. However, all dystonias feature excessive muscle activation that is associated with hyperactivity in multiple motor areas associated with movement activation. Electrophysiological studies of dystonia patients confirms a pattern of hyperactivation in cortex. Healthy individuals show low beta oscillations (~15–20 Hz) in motor cortical local field potential (LFP). This beta is reduced in amplitude and synchrony during movement (Jasper and Penfield, 1949; Pfurtscheller and Aranibar, 1979; Crone et al., 1998; Miller et al., 2007). In dystonia patients, motor cortex shows increases in neuronal activity levels (Nobrega et al., 1995; Pratt et al., 1995), with relatively high beta amplitude and high functional connectivity at the beta frequency (Schnitzler and Gross, 2005; Jin et al., 2011). There is also excessive neural synchrony both at rest and in certain phases of movement (Toro et al., 1994; Kristeva et al., 2005; Mallet et al., 2008; Crowell et al., 2012).

Some dystonias, in common with several other movement disorders, are thought to have their origin in the basal ganglia. Other dystonias, such as those associated with cerebral palsy and with movement overuse, probably have a strong cortical component. In all cases, however, the interconnections of brain motor systems makes it clear that multiple brain areas will be “in the loop” of abnormal activity. Following some primary insult or insults to a brain area, a secondarily-involved brain area will contribute further to the disorder by reacting to the alterations in input activity through its own homeostatic responses. In some cases these homeostatic changes may be compensatory so as to reduce the severity of the symptoms. However, in other cases, plasticity may actually exacerbate the abnormal movements (Sanger et al., 2003; Neychev et al., 2008; Casellato et al., 2014).

There are at least two, and perhaps more, cerebello-thalamo-striato-cortical loops that play a role in movement disorders. There may also be additional contributions from still longer loops involving recurrent connections from spinal cord or from muscle spindles. One or more of these sites may have associated pathology. Regardless of the locus of primary pathology, multiple sites are potential targets where therapy could interrupt pathophysiological dynamics. Currently, brain pharmacotherapy often fails and patients are treated with botulinum toxin to partially paralyze muscles by blocking nicotinic cholinergic transmission at the affected muscle. Another treatment is deep brain stimulation using implanted electrodes. In this paper, we take two or three steps back from the level of muscle treatment by proposing interventions at the level of motor cortex.

Complex multifocal diseases may require complex multitarget treatments (Viayna et al., 2013). In the context of brain disease, multitarget therapy can hit multiple brain regions or multiple receptors in a region or both. High-level models that include many brain areas can assist in understanding how different brain areas contribute to a disorder (Sanger and Merzenich, 2000; Sanger, 2003; Hendrix and Vitek, 2012; Kerr et al., 2013). However, these models typically lack biological detail, making them unsuitable for assessing the impact of specific pharmacological manipulations. Detailed models are not yet elaborated to the point of handling multiple brain areas but do provide the details needed to assess pharmacological intervention more directly.

Single agent treatments for disease are traditionally tested *in vitro* or *in vivo*. As noted above, single agent treatments for dystonia have not had much success. There is, however, the potential for success with multitarget drug cocktails that could target multiple locations in the brain, or multiple drug receptor targets at a single location, or both (Delnooz and van de Warrenburg, 2012). Due to combinatorial explosion, evaluating combinations of drugs in different dosages in this way can not be readily done in tissue and is most feasible *in silico*(Viceconti et al., 2008; Kohl and Noble, 2009; Lytton et al., 2014; Action, 2016; Viceconti et al., 2016). In this study, we use our detailed multiscale model of primary motor cortex to assess potential multitarget pharmacological therapies for treatment of dystonia. The model contains 6 cortical layers with multiple classes of excitatory and inhibitory neurons, using wiring based on mouse microconnectomic data (Shipp, 2005; Weiler et al., 2008; Kiritani et al., 2012; Hooks et al., 2013). Excitatory neurons contain intracellular molecular mechanisms that contribute to persistent activity and hyperexcitability (Neymotin et al., 2016). These mechanisms include endoplasmic reticulum associated calcium stores released by activation of IP_3R_ s, and ryanodine receptors, both with affinity for caffeine, an agent that can exacerbate dystonia symptoms (Richter and Hamann, 2001). Plasma membrane calcium, sodium, and potassium channels also contribute to cellular excitability.

Since our model does not include spinal cord and muscle, we defined dystonia pathology as a state of cortical hyperactivation characterized by increased beta oscillations with excessive and hypersynchronous firing in layer 5 corticospinal neurons. These layer 5 neurons project downward to brainstem and spinal cord, and their sustained firing would lead to the increased muscle contractions of dystonia. We distinguished the hyperexcitability of dystonia from the still greater hyperexcitability of a seizure by excluding simulations that showed higher levels of activity with higher frequency oscillation and a strong tendency to “latch-up” through multicell depolarization blockade (Lytton and Omurtag, 2007). Classification in 11-dimensional space demonstrated that we could identify different regions in parameter space for these different states—baseline normal, dystonia, epileptiform—and predict pharmacological combinations that would lead from pathology back to the physiological activity state. As in our previous investigations of epilepsy (Lytton and Omurtag, 2007), we found multiple parameter combinations that were consistent with the pathological state, as well as multiple parameter combinations to produce our baseline physiological state. Such parameter degeneracy is typical of complex neural systems (Edelman and Gally, 2001; Golowasch et al., 2002).

## 2. Materials and methods

**Figure 1:**
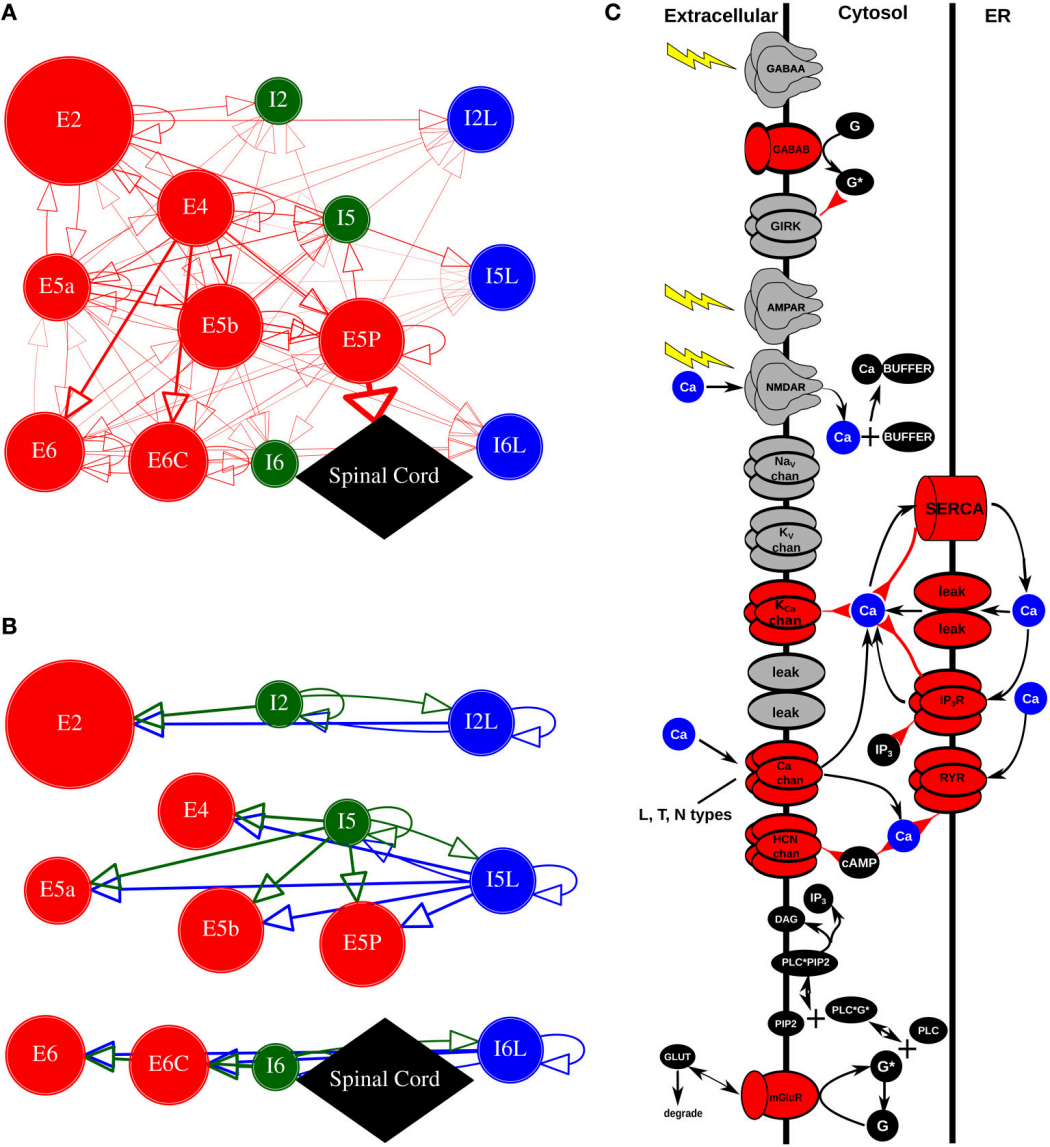
**Model schematics. (A,B)** Motor cortex architecture. Circles represent neuronal populations (red: excitatory cells; green: fast-spiking interneurons; blue: low-threshold firing interneurons). Circle size denotes number of cells in population. Lines (with arrows) indicate connections between the populations. Thickness of lines proportional to synaptic weights. E cells synapse with AMPAR/NMDARs; I cells synapse with GABA_A_ R / GABA_B_ Rs. Circles with self-connects denotes recurrent connectivity. **(A)** Excitatory connections. E5P projects to spinal cord (not modeled). **(B)** Inhibitory connections. **(C)** Chemical signaling in pyramidal cells showing fluxes (black arrows) and second- (and third- etc) messenger modulation (red back-beginning arrows). We distinguish membrane-associated ionotropic and metabotropic receptors and ion channels involved in reaction schemes in red (in reality, it is likely that almost every membrane-bound protein is modulated). External events are represented by yellow lightning bolts—there is no extracellular diffusion; the only extracellular reaction is glutamate binding, unbinding, and degradation on mGluR1 after an event. Ca^2+^ is shown redundantly in blue—note that there is only one Ca^2+^ pool for extracellular, 1 pool for cytoplasmic, and 1 pool for ER (PLC, phospholipase C; DAG, diacyl-glycerol; cAMP, cyclic adenosine monophosphate; PIP_2_, phosphatidylinositol 4,5-bisphosphate). Adapted from Figure 1 of Neymotin et al. (2016).

Network simulations consisted of 1715 reduced compartmental cell models with single compartments for inhibitory cells and five compartments for pyramidal cells, arrayed by layer with connectivity taken from experimental results on motor cortex (Weiler et al., 2008; Figures 1A,B). Parallel-conductance electrophysiological simulation in the pyramidal cells was complemented by chemophysiological simulation focused on Ca^2+^ handling, based on our prior models (Neymotin et al., 2015, 2016; Figure 1C).

Simulations were run in the NEURON (version 7.4) simulation environment (Carnevale and Hines, 2006) utilizing the reaction-diffusion (RxD) Python module (McDougal et al., 2013a,b) and NMODL (Hines and Carnevale, 2000). Two seconds of simulation time took ~3 min using 24 nodes on a Linux cluster with parallel NEURON, run with a fixed time-step of 0.1 ms. The full model is available on ModelDB (https://senselab.med.yale.edu/ModelDB/ShowModel.cshtml?model=189154).

We briefly describe the scales of the multiscale model from smaller to larger in the following sections (Table 1). For more details, readers are referred to our previous papers (Neymotin et al., 2015, 2016).

**Table 1:** **Summary of model**.

**Property**	**Description**
Populations	13; 7 E and 6 I, corresponding to layer 2/3, 4, 5A, 5B, and 6 of M1
Topology	3D with cortical depth (y) based on M1 laminar distribution, horizontal location (x,z) randomly distributed
Connectivity	Probability of connection and weight depends on layer and cell type
Neuron model	Multichannel multicompartment (E cells also RxD mechanisms)
Synapse model	AMPA, NMDA, GABA_A_, GABA_B_, mGluR
Plasticity	–
Input	Independent random Poisson spike trains with fixed rate depending on cell type/synapse
Measurements	Membrane potential, spiking activity, synchronization, firing vector correlations

*E (I) denote excitatory (inhibitory) neurons. No plasticity modeled (Table format based on Nordlie et al., 2009)*.

### 2.1. Intracellular molecular scale

Our *Ca*^2+^ dynamics (Figure 1C), are based on (Neymotin et al., 2016). We modeled a one-dimensional RxD system of intracellular neuronal Ca^2+^ signaling in all compartments of neocortical pyramidal (PYR) neurons. Within each compartment, we modeled cytosolic and endoplasmic reticulum (ER) sub-compartments by using a fractional volume for each.

IP_3_ was produced through a reaction sequence initiated by glutamate binding to the metabotropic glutamate receptor (mGluR), based on a reaction scheme developed by Ashhad and Narayanan (2013) (ModelDB #150551). IP_3_ diffused outward from the synapse location and decayed following first-order kinetics (τ_IP_3__ of 1 s). Baseline mGluR synaptic weight was normalized to represent the increase in the amount of glutamate bound to mGluR. Extracellular glutamate did not diffuse but was represented by a local Glu value that was incremented in response to an event delivered due to a presynaptic spike. Glu showed bind/unbind kinetics on mGluR and was eliminated by first-order degradation (lower left of Figure 1C).

The ER Ca^2+^ model involves IP_3_ receptors (IP_3R_ s), ryanodine receptors (RYR) (Sneyd et al., 2003), SERCA pumps, and a Ca^2+^ leak. IP_3R_ dynamics involved a slow Ca^2+^ inactivation binding site state (De Young and Keizer, 1992; Li and Rinzel, 1994). The SERCA pump is a pump rather than a channel and so is modeled with Hill-type dynamics. Calcium buffering followed $Ca+B\;\overset{5}{\underset{9.5\cdot 10^{-4}}{⇌}}CaB$ where *B* is diffusible buffer with diffusion coefficients D = 0.043 μ*m*^2^∕*ms* for both *B* and *CaB*, about half the rate of Ca^2+^ diffusion (Anwar et al., 2012). Calcium extrusion across the plasma membrane was modeled by first-order decay with τ_*ex*_ = 5 ms.

### 2.2. Synapses

AMPA/NMDA synapses were modeled by standard NEURON double-exponential mechanisms (Table 2). All excitatory projections were mixed AMPA (rise,decay τ: 0.05, 5.3 ms) and NMDA (rise,decay τ: 15, 150 ms). NMDARs were scaled by 1∕(1+0.28 · *Mg* · *exp*(−0.062 · *V*)); *Mg* = 1*mM* (Jahr and Stevens, 1990). 13% of *I*_*NMDA*_ was carried by Ca^2+^ (Spruston et al., 1995). AMPA and NMDA receptors had reversal potentials of 0 mV.

**Table 2:** **Summary of synapse models used to connect neurons**.

**Label**	**Description**
AMPA	Double exponential
NMDA	Double exponential with voltage dependence
GABA_A_	Double exponential
GABA_B_	2nd messenger connectivity to a G protein-coupled inwardly-rectifying potassium channel (GIRK)
mGluR	2nd messenger signaling producing IP_3_

Inhibitory synapses were mediated by GABA_A_ and GABA_B_ receptors. GABA_A_ synapses were modeled with a double-exponential mechanism. The GABA_B_ synapse had second messenger connectivity to a G protein-coupled inwardly-rectifying potassium channel (GIRK). LTS cells connected to apical dendrites of PYR cells using GABA_A_ receptors (GABA_A_ R; rise,decay τ: 0.2, 20 ms) and GABA_B_ receptors (GABA_B_ R) and onto somata of FS and other LTS cells with GABA_A_ Rs (rise,decay τ: 20, 40 ms). GABA_A_ Rs had reversal potentials of −80 mV, and GABA_B_ Rs −95 mV. GABA_B_ Rs provide longer-lasting activation compared to GABA_A_ Rs.

### 2.3. Cell scale

The network consisted of pyramidal cells (PYR; 3 apical dendrite compartments, 1 basal dendrite compartment, 1 somatic compartment), fast spiking soma-targeting interneurons (FS; one compartment), and dendrite-targeting low-threshold spiking interneurons (LTS; one compartment; Wang and Buzsaki, 1996; Wang, 2002; Monyer and Markram, 2004; Bartos et al., 2007; Neymotin et al., 2011a,b; Tables 3, 4). Reaction-diffusion mechanisms (Ca^2+^,IP_3_,buffer) were restricted to the PYR cells in this network. Properties of pyramidal neurons in the different layers were identical except for apical dendrite length which is longer in deep pyramidal neurons than in superficial (Hay et al., 2011; Castro-Alamancos, 2013): 900 μm in L5-6; 450 μm in L2/3 and L4.

**Table 3:** **Summary of neuron models**.

**Label**	**Description**
Dynamics	Multichannel compartmental Hodgkin-Huxley (plus RxD mechanisms)
Compartments	E: 5 (soma, basal dendrite, 3 apical dendrites)
Compartments	I: 1 (soma)
Ion channels	E: leak, *Na*_*f*_, *K*_*dr*_, *K*_*a*_, *K*_*D*_, *K*_*M*_, HCN, CaL, CaN, CaT, *SK*, *BK*
Ion channels	I: leak, *Na*_*f*_, *K*_*dr*_, HCN, CaL, *K*_*M*_
RxD molecules	E: Ca^2+^, IP_3_, *B* (Ca^2+^ buffer), *CaB* (Ca^2+^-bound Ca^2+^-buffer)
RxD compartments	E: endoplasmic reticulum, cytosol
RxD channels	E: leak, RyR, IP_3R_, SERCA

*E (I) denote excitatory (inhibitory) neurons. Reaction-diffusion (RxD) mechanisms/ compartments described more fully in intracellular scale*.

**Table 4:** **Network Population, including normalized and nominal cortical depth range (ynormRange, yRange, neuron density, and number of cells)**.

**Label**	**Description**	**ynormRange**	**yRange (um)**	**Density (cells/mm^3^)**	**numCells**
E2	Layer 2/3 PYR IT excitatory neurons	0.12–0.31	160–420	80,000	300
E4	Layer 4 PYR IT excitatory neurons	0.31–0.42	420–570	80,000	173
I2	Layers 2/3 FS interneurons	0.12–0.31	160–420	10,000	37
I2L	Layers 2/3 LTS interneurons	0.12–0.31	160–420	10,000	37
E5a	Layer 5a PYR IT excitatory neurons	0.42–0.52	570–700	80,000	150
E5b	Layer 5b PYR IT excitatory neurons	0.52–0.77	700–1040	40,000	196
E5P	Layer 5b PYR PT excitatory neurons	0.52–0.77	700–1040	40,000	196
I5	Layers 4 and 5 FS interneurons	0.31–0.77	420–1040	10,000	89
I5L	Layers 4 and 5 LTS interneurons	0.31–0.77	420–1040	10,000	89
E6	Layer 6 PYR IT excitatory neurons	0.77–1.0	1040–1350	40,000	179
E6C	Layer 6 PYR CT excitatory neurons	0.77–1.0	1040–1350	40,000	179
I6	Layer 6 FS interneurons	0.77–1.0	1040–1350	10,000	45
I6L	Layer 6 LTS interneurons	0.77–1.0	1040–1350	10,000	45

*PYR, pyramidal; IT, intratelencephalic; PT, pyramidal tract; CT, corticothalamic; FS, fast spiking, LTS, low-threshold spiking*.

Voltage-gated ionic current models were based on prior models of our own and others (McCormick and Huguenard, 1992; Migliore et al., 2004; Stacey et al., 2009; Neymotin et al., 2011b,a, 2013). Voltage sensitive channels generally followed the Hodgkin-Huxley parameterization, whereby ẋ = (*x*_∞_ − *x*)∕τ_*x*_ (*x* = *m* for activation particle and *h* for inactivation particle). Steady-state *x*_∞_ and time constant τ_*x*_ are either related to channel opening α(*V*) and closing kinetics β(*V*) as *x*_∞_ = α∕(α + β), τ_*x*_ = 1∕(α + β), or are directly parameterized: *x*_∞_(*V*), τ_*x*_(*V*). Kinetics for channels were scaled by Q_10_ from an experimental temperature (where available) to simulation temperature of 37°C. Q_10_ = 3 was used when no experimental value was available. All cells contained leak current, transient sodium current *I*_*Na*_, and delayed rectifier current *I*_*K*−*DR*_, to allow for action potential generation. Additionally, PYR cells contained in all compartments *I*_*K*−*A*_, *I*_*K*−*M*_ providing firing-rate adaptation (McCormick et al., 1993). Pyramidal cells also had *I*_h_, voltage-gated calcium channels (VGCCs) in all compartments: *I*_*L*_, *I*_*T*_, *I*_*N*_ (Kay and Wong, 1987; McCormick and Huguenard, 1992; Safiulina et al., 2010; Neymotin et al., 2015), and SK and BK calcium-activated potassium currents (*I*_*KCa*_). LTS cells contained *I*_*L*_, non-Ca^2+^-dependent *I*_h_, SK, and Ca^2+^ decay.

HCN channels in different cell types have somewhat different voltage dependence and different kinetics (Hagiwara and Irisawa, 1989; Schwindt et al., 1992; Chen et al., 2001; Wang et al., 2002; Robinson and Siegelbaum, 2003). The hyperpolarization-activated HCN current *I*_*h*_ used in pyramidal cells was modeled with second messenger and calcium dependence taken from Winograd et al. (2008) (ModelDB #113997), and modified to provide the faster voltage-sensitivity time constants found in cortex (Harnett et al., 2015), and provides PYR cells longer-lasting firing activity via augmentation of the HCN channel conductance. The mechanism for Ca^2+^ regulation of HCN channels in PYR cells in Winograd et al. (2008) is modeled empirically in order to produce the relationship between cytosolic Ca^2+^ levels and *I*_h_ activation without simulating the details of Ca^2+^ effects on adenyl cyclase (see schematic for *HCN chan* in Figure 1C).

*g*_*h*_ was 0.0025 S/*cm*^2^ in PYR soma, basal dendrites and exponentially-increasing in apical dendrites with distance from soma with 325 μm space constant, hence *e*-fold augmented at 325 microns as described by Kole et al. (2006). Apical dendrite *I*_*K*−*DR*_, *I*_*K*−*A*_, *I*_*K*−*M*_ density also increased with the same length constant, based on data showing HCN and Kv channel colocalization (Harnett et al., 2015, 2013).

### 2.4. Network scale

The network consisted of 1715 cells (Table 4). The network contained 157,507 synapses for an overall connection density of ~5% (see **Table 6**). PYR cells synapsed onto each-other's dendrites. PYR-to-PYR synaptic locations on the dendrite were randomized between basal and apical compartments (Markram et al., 1997). PYR cells synapsed onto somata of FS and LTS cells (single-compartment models).

Neuronal populations were arranged by cortical layer based on our prior models (Neymotin et al., 2011a,c; Chadderdon et al., 2014; Neymotin et al., 2016), with additional data from direct measurements from mouse motor cortex (Shipp, 2005; Weiler et al., 2008; Kiritani et al., 2012; Hooks et al., 2013), and recent experiments which demonstrate a thin layer 4 in mouse motor cortex (Yamawaki et al., 2014). The network consisted of 13 populations of 3 excitatory cell types, intratelencephalic (IT), pyramidal-tract (PT), and corticothalamic (CT), and 2 inhibitory cell types, low-threshold spiking (LTS) and fast-spiking (FS). These were distributed across cortical layers 2/3, 4, 5a, 5b, and 6 (Harris and Shepherd, 2015), with cell numbers for each population based on estimated cell densities and volume (Table 4). The volume of each population was calculated assuming a horizontal area (x and z dimensions) of 120 × 120 μm, and a layer-dependent cortical depth range (y dimension).

Connection probabilities *p*_*ij*_ (Tables 5, 6) of presynaptic excitatory populations were dependent on pre- and pothst-synaptic type and layer. For presynaptic inhibitory populations, connection probabilities inversely scaled based on distance $p_{ij}={\bar{p}}_{ij}\cdot \exp\left(-\sqrt{\left(dx^{2}+dz^{2}\right)}∕15\right)$, in x, z plane perpendicular to the y-direction of layering. Connection probabilities and weights are based on data from rodent motor cortex mapping (Weiler et al., 2008; Lefort et al., 2009; Anderson et al., 2010; Fino and Yuste, 2011; Apicella et al., 2012; Kiritani et al., 2012). Individual neurons were placed randomly with uniform distribution. Weights from E cells displayed in Table 6 are for the AMPA synapses, with colocalized NMDA weights at 10% of AMPA weights. Synaptic delays were randomized between 1.8 and 5 ms with additional delay based on distance.

**Table 5:** **Summary of network connectivity rules**.

**Property**	**Description**
E to E	*p*_*ij*_, *w*_*ij*_ dependent on pre-/post-synaptic cell type/layer
E to I	*p*_*ij*_, *w*_*ij*_ dependent on pre-synaptic cell layer, and post-synaptic cell type/layer
I to E/I	*p*_*ij*_ decreases exponentially with x,z plane distance between pre-/post-synaptic neurons; fixed *w*_*ij*_
All delays	Randomized 1.8–5 ms with additional delay based on distance

*p_ij_ denotes probability of connection between type i and j; w_ij_ denotes weight. Parameters by pre- and post-synaptic type in Table 6*.

**Table 6:** **Network Connectivity Parameters**.

**Pre**	**Post**	***${\bar{p}}_{ij}$***	***w_*ij*_* (nS)**	**Pre**	**Post**	***${\bar{p}}_{ij}$***	***w_*ij*_* (nS)**	**Pre**	**Post**	***${\bar{p}}_{ij}$***	***w_*ij*_* (nS)**
I2L	I2L	1.00	0.150	I2L	I2	1.00	0.150	I2L	E2	1.00	0.225
I2L	E2	1.00	1.688	I2	I2L	1.00	0.150	I2	I2	1.00	0.150
I2	E2	1.00	0.225	E2	I2L	0.19	0.117	E2	I2	0.19	0.117
E2	E2	0.15	0.160	E2	E4	0.11	0.092	E2	I5L	0.22	0.151
E2	I5	0.02	0.017	E2	E5a	0.05	0.126	E2	E5b	0.01	0.111
E2	E5P	0.07	0.111	E4	I2L	0.02	0.054	E4	I2	0.02	0.054
E4	E2	0.05	0.184	E4	E4	0.15	0.160	E4	I5L	0.03	0.018
E4	I5	0.19	0.162	E4	E5a	0.04	0.160	E4	E5b	0.01	0.225
E4	E5P	0.01	0.225	E4	I6L	0.02	0.066	E4	I6	0.02	0.066
E4	E6C	0.00	0.477	E4	E6	0.00	0.477	I5L	E4	1.00	0.225
I5L	E4	1.00	1.688	I5L	I5L	1.00	0.150	I5L	I5	1.00	0.150
I5L	E5a	1.00	0.225	I5L	E5a	1.00	1.688	I5L	E5b	1.00	0.225
I5L	E5b	1.00	1.688	I5L	E5P	1.00	0.225	I5L	E5P	1.00	1.688
I5	E4	1.00	0.225	I5	I5L	1.00	0.150	I5	I5	1.00	0.150
I5	E5a	1.00	0.225	I5	E5b	1.00	0.225	I5	E5P	1.00	0.225
E5a	I2L	0.02	0.054	E5a	I2	0.02	0.054	E5a	E2	0.04	0.131
E5a	E4	0.03	0.104	E5a	I5L	0.03	0.018	E5a	I5	0.19	0.162
E5a	E5a	0.18	0.143	E5a	E5b	0.01	0.208	E5a	E5P	0.02	0.208
E5a	I6L	0.02	0.066	E5a	I6	0.02	0.066	E5a	E6C	0.01	0.081
E5a	E6	0.01	0.081	E5b	I2L	0.02	0.054	E5b	I2	0.02	0.054
E5b	E2	0.02	0.059	E5b	E4	0.03	0.043	E5b	I5L	0.03	0.018
E5b	I5	0.19	0.162	E5b	E5a	0.05	0.080	E5b	E5b	0.18	0.171
E5b	E5P	0.04	0.171	E5b	I6L	0.02	0.066	E5b	I6	0.02	0.066
E5b	E6C	0.02	0.122	E5b	E6	0.02	0.122	E5P	I2L	0.02	0.054
E5P	I2	0.02	0.054	E5P	I5L	0.03	0.018	E5P	I5	0.19	0.162
E5P	E5P	0.18	0.171	E5P	I6L	0.02	0.066	E5P	I6	0.02	0.066
I6L	I6L	1.00	0.150	I6L	I6	1.00	0.150	I6L	E6C	1.00	0.225
I6L	E6C	1.00	1.688	I6L	E6	1.00	0.225	I6L	E6	1.00	1.688
I6	I6L	1.00	0.150	I6	I6	1.00	0.150	I6	E6C	1.00	0.225
I6	E6	1.00	0.225	E6C	I5L	0.02	0.037	E6C	I5	0.02	0.037
E6C	E5a	0.03	0.034	E6C	E5b	0.03	0.077	E6C	E5P	0.03	0.077
E6C	I6L	0.02	0.080	E6C	I6	0.02	0.080	E6C	E6C	0.03	0.133
E6C	E6	0.02	0.133	E6	I5L	0.02	0.037	E6	I5	0.02	0.037
E6	E5a	0.03	0.034	E6	E5b	0.03	0.077	E6	E5P	0.03	0.077
E6	I6L	0.02	0.080	E6	I6	0.02	0.080	E6	E6C	0.02	0.133
E6	E6	0.03	0.133								

*${\bar{p}}_{ij}$ and w_ij_ are distance-independent probability of connections from Pre to Post neuronal types and synaptic weights, respectively*.

Background activity was simulated by excitatory and inhibitory synaptic inputs following a Poisson process, sent to all cells, representing ongoing drive from other cortical areas and other inputs. These inputs were selected to maintain low-frequency firing of neurons within the model, which would not fire otherwise, due to small network size and the requirement for multiple synaptic inputs to trigger a postsynaptic spike (Neymotin et al., 2011a). The strength of these background inputs was not based on the full source of inputs from all upstream brain areas, since these inputs are not completely mapped.

### 2.5. Simulation variations

We ran over 5800 simulations, randomly varying each of the following parameters using an independent normal distribution: 1. E neuron mGluR density (mGluR); 2. E neuron ER RYR density (RYR); 3. E and I neuron HCN channel density; 4. E and I neuron fast Na^+^ channel density (*Na*_*f*_); 5. E neuron *K*_*dr*_ channel density; 6. E neuron *K*_*a*_ channel density; 7. E neuron *K*_*D*_ channel density; 8. E neuron *K*_*M*_ channel density; 9. E neuron *SK* calcium-activated potassium channel density; 10. E neuron *BK* calcium-activated potassium channel density; 11. E and LTS neuron voltage-gated calcium channel (VGCC) density.

Means and standard deviations differed for the different parameters and were selected to allow each simulation to maintain activity. A subset of the simulations was used for the analyses described (Table 7).

**Table 7:** **Parameter ranges (average ± standard deviation) for all simulations (*n* = 5867), active simulations (*n* = 4341), latch-up simulations (*n* = 1077), active/non-Latch-up simulations (*n* = 3264), physiological simulations (*n* = 65), and dystonia simulations (*n* = 65)**.

**Parameter**	**All**	**Active**	**Latch-up**
*mGluR*	8.06 ± 6.44	8.02 ± 6.43	8.04 ± 6.34
*RYR*	108.54 ± 86.99	109.74 ± 86.74	112.03 ± 86.98
*HCN*	0.0025 ± 0.0003	0.0026 ± 0.0002	0.0026 ± 0.0002
*Na*_*f*_	0.0809 ± 0.0081	0.0829 ± 0.0074	0.0856 ± 0.0072
*K*_*dr*_	0.0209 ± 0.0053	0.0202 ± 0.0052	0.0216 ± 0.0054
*K*_*a*_	0.3000 ± 0.0150	0.2977 ± 0.0147	0.2967 ± 0.0144
*K*_*d*_	0.0009 ± 0.0002	0.0008 ± 0.0002	0.0008 ± 0.0002
*K*_*m*_	1.002e-05 ± 2.48e-06	1e-05 ± 2.49e-06	1.001e-05 ± 2.51e-06
*SK*	0.0001 ± 6.163e-05	0.0001 ± 6.18e-05	0.0001 ± 6.296e-05
*BK*	0.0030 ± 0.0015	0.0030 ± 0.0015	0.0031 ± 0.0015
*VGCC*	0.0052 ± 0.0035	0.0053 ± 0.0035	0.0051 ± 0.0035
**Parameter**	**Active/Non-Latch-up**	**Physiological**	**Dystonia**
*mGluR*	8.02 ± 6.45	8.42 ± 6.54	8.12 ± 5.74
*RYR*	108.99 ± 86.66	105.1 ± 82.9	116.64 ± 77.11
*HCN*	0.0026 ± 0.0002	0.0026 ± 0.0002	0.0026 ± 0.0003
*Na*_*f*_	0.0820 ± 0.0073	0.0787 ± 0.0053	0.0879 ± 0.0076
*K*_*dr*_	0.0198 ± 0.0051	0.0226 ± 0.0041	0.0195 ± 0.0054
*K*_*a*_	0.2981 ± 0.0148	0.3029 ± 0.0144	0.2992 ± 0.0136
*K*_*d*_	0.0008 ± 0.0002	0.0008 ± 0.0002	0.0008 ± 0.0002
*K*_*m*_	1e-05 ± 2.48e-06	1.034e-05 ± 2.42e-06	1.021e-05 ± 2.81e-06
*SK*	0.0001 ± 6.135e-05	0.0001 ± 6.797e-05	0.0001 ± 6.604e-05
*BK*	0.0030 ± 0.0015	0.0034 ± 0.0013	0.0025 ± 0.0015
*VGCC*	0.0054 ± 0.0035	0.0058 ± 0.0032	0.0046 ± 0.0031

*Plasma membrane ion channel conductance density values are in S/cm^2^. mGluR and RYR density are in arbitrary units used to scale channel conductance*.

We ran simulations with initial calcium concentration in the ER set to 1.25 mM (Bygrave and Benedetti, 1996), to mimic the effects of ER calcium priming via prior excitatory synaptic stimulation (Ross et al., 2005; Hong and Ross, 2007; Fitzpatrick et al., 2009; Neymotin et al., 2016).

We categorized the simulations into distinct groups by noting major differences in activity across parameter sets (Table 8). From the full set of 5867 simulations, 1505 did not display any firing due to random variations in ion channel densities which led to low neuronal activity (Table 7). The remaining 4341 simulations were *Active* due to higher neuronal activity, e.g., partially caused by the higher average *Na*_*f*_ density in these simulations. Of these 4341 Active simulations, 1077 exhibited epileptic *latch-up* dynamics—periods of intense activity which led to *depolarization blockade* (Na^+^ channel inactivation; Lytton and Omurtag, 2007). These periods where neurons did not fire lasted 200–300 ms (gaps between E5P spikes: E5P gap in Table 8). We categorized the top and bottom 2nd percentile of the Active/non-latch-up simulations ranked by E5P firing rate into dystonia (*n* = 65) and physiological (*n* = 65) sub-sets. We used E5P firing rate as a criterion for dystonia classification because E5P neurons project downward to brainstem spinal cord, and sustained overactive E5P firing can lead to the tonic muscle contractions symptomatic of dystonia.

**Table 8:** **Dynamic measures (average ± standard deviation) for All simulations (*n* = 5867), Active simulations (*n* = 4341), Latch-up simulations (*n* = 1077), Active/Non-Latch-up (*n* = 3264), physiological simulations (*n* = 65), and dystonia simulations (*n* = 65)**.

**Dynamic measure**	**All**	**Active**	**Latch-up**	**Active/non-latch-up**	**Physiological**	**Dystonia**
E5a rate (Hz)	0.65 ± 0.52	0.88 ± 0.41	1.09 ± 0.37	0.81 ± 0.40	1.34 ± 0.51	0.85 ± 0.39
E5b rate (Hz)	1.68 ± 1.21	2.27 ± 0.79	2.45 ± 0.66	2.22 ± 0.82	1.18 ± 0.28	3.74 ± 2.08
E5P rate (Hz)	7.10 ± 5.62	9.59 ± 4.32	7.77 ± 2.68	10.19 ± 4.59	1.77 ± 0.26	22.59 ± 2.67
I5 rate (Hz)	11.46 ± 6.99	15.49 ± 1.89	15.14 ± 1.28	15.61 ± 2.04	11.47 ± 0.72	17.67 ± 0.90
I5L rate (Hz)	5.81 ± 3.71	7.85 ± 1.61	7.13 ± 1.19	8.09 ± 1.66	5.37 ± 0.76	13.42 ± 1.82
E5P synchrony	0.35 ± 0.25	0.47 ± 0.16	0.47 ± 0.12	0.47 ± 0.17	0.07 ± 0.06	0.75 ± 0.05
E5P MUA freq. (Hz)	14.78 ± 8.91	19.97 ± 1.94	19.72 ± 1.79	20.05 ± 1.99	20.91 ± 3.11	20.55 ± 0.88
E5P MUA amp. (AU)	83.0 ± 100.1	112.1 ± 101.4	59.8 ± 48.1	129.4 ± 108.1	1.9 ± 1.2	527.0 ± 161.7
E5P MUA beta amp. (AU)	21.0 ± 26.4	28.3 ± 27.1	15.9 ± 11.9	32.4 ± 29.4	0.8 ± 0.7	111.2 ± 64.9
E5P gap	79.13 ± 70.95	106.94 ± 61.87	190.23 ± 32.94	79.46 ± 41.10	62.77 ± 24.85	21.75 ± 32.11
E5P FV sim	0.20 ± 0.14	0.27 ± 0.09	0.24 ± 0.06	0.28 ± 0.09	0.13 ± 0.03	0.44 ± 0.08

*E5P gap measures number of 300 ms gaps between individual E5P neuron firing times; E5P MUA amplitude and E5P MUA beta amplitude in arbitrary units; E5P FV sim measures similarity between E5P population firing rate vectors using average pairwise Pearson correlation*.

### 2.6. Data analysis

We formed multiunit activity (MUA) time-series, which count the number of spikes in each bin (10 ms) for a given population. To calculate neuronal population rhythms, we took the power spectral density (PSD) of the mean-subtracted MUA time-series; we then calculated the peak frequencies and amplitudes in the PSD. We used the average Kendall's τ non-parametric rank-correlation coefficient (Kendall, 1938; Knight, 1966) between pairs of neuron binned spike train time-series for calculating the synchronization of populations of neurons (denoted population-synchrony). Kendall's τ non-parametric rank correlation, defined as:

$$\tau =\frac{n_{c}-n_{d}}{\frac{1}{2}n(n-1)},$$

is used with these data. Kendall's τ is a normalized difference between concordant (*n*_*c*_) and discordant pairs (*n*_*d*_); ties are taken into account by the normalizing term, $\frac{1}{2}n\left(n-1\right)$, which represents the total number of ordered pairs in the time-series. We used the Python scikit-learn library (Pedregosa et al., 2011) for performing principal component analysis (PCA) and support-vector machine (SVM) classification (Cortes and Vapnik, 1995; Orrù et al., 2012). Dystonia and physiological simulation classes were characterized on the basis of layer 5 corticospinal pyramidal neuron (E5P) firing rates. The clearest examples of both classes (bottom/top 2nd percentiles as physiological/dystonia classes) were used for the majority of the analyses described in the Results (**Figures 3–8**). The NuSVC variant of SVMs was used to classify physiological and dystonia simulations and to find which simulation parameters contributed the most to classification accuracy. SVM inputs were vectors consisting of normalized parameter values. Each of these input vectors was labeled into either of two distinct binary classes: physiological (0) or dystonia (1). SVM parameters, including kernel type (linear, polynomial, radial-basis function), γ, tolerance, ν, and polynomial degree were selected using a grid search with *N*-fold cross validation run 10 times for each combination of parameters. SVM classification accuracy surpassed the accuracy of other machine learning methods, including logistic regression (not shown). Figures were drawn with Matplotlib (Hunter, 2007).

## 3. Results

### 3.1. Simulation overview

We ran over 5800 network simulations, randomizing 11 ion channel/receptor densities independently. A typical 2 s simulation took ~3 min using 24 cores on Linux with parallel NEURON. After running simulations, we calculated neuronal population firing rates, synchronization, and power spectra.

### 3.2. Characterization of dystonia pathophysiology

**Figure 2:**
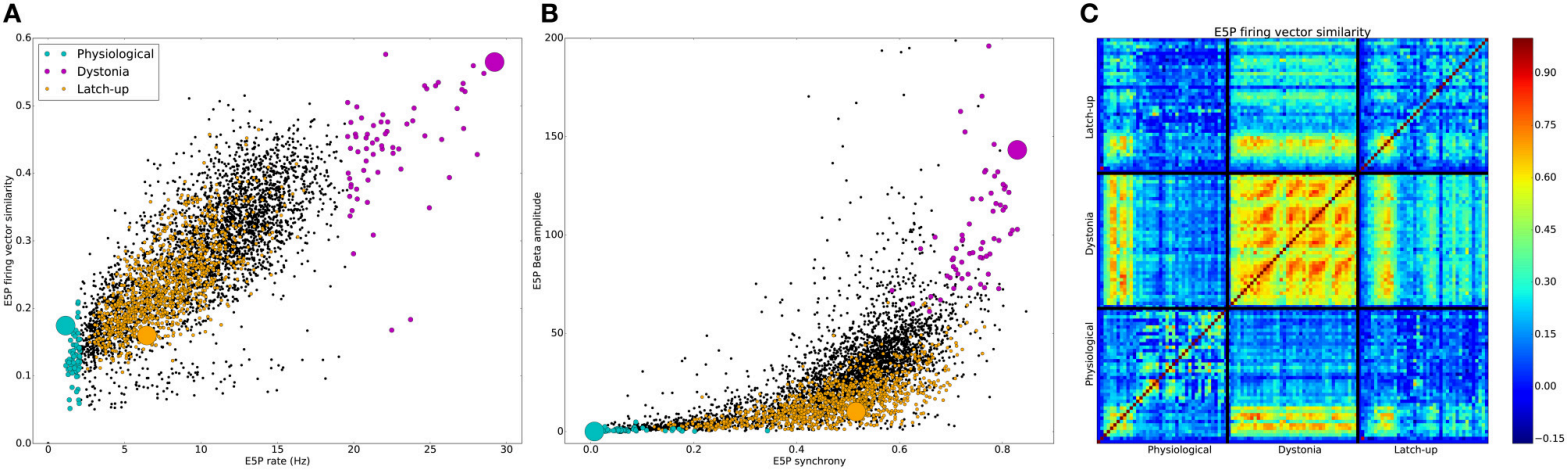
**Distinct dynamics in in physiological, dystonia, and latch-up simulations**. **(A)** Average E5P firing rate vector (FV) similarity vs. average E5P firing rate for individual simulations. **(B)** E5P MUA Beta oscillation amplitude vs. E5P synchrony for individual simulations. **(A,B)** [light blue: physiological, purple: dystonia, orange: epileptiform, black: remaining Active simulations, large circles represent simulations shown in **(C)** and Figure 3]. **(C)** Pearson correlations between all pairs of E5P FVs. Solid black lines demarcate FVs from example physiological, dystonia, and epileptiform simulations. All FVs used 50 ms intervals, forming 40 FVs per 2 s of simulation.

Simulations were grouped into physiological and pathological based on differences in firing patterns (Table 8, Figure 2). 1505 of 5867 simulations produced no activity. The remaining simulations were characterized as physiological or pathological. Pathological simulations showed increased activity. High activity alternating with latch-up condition was defined as an epileptiform simulation with periods of >200 ms of depolarization blockade across multiple cells (1077 simulations). 1077 simulations were classified as epileptiform based on activity latch-up resulting in sustained periods. The different classes of simulations formed distinct clusters in multiple slices of excitatory corticospinal (ESP) activity feature-space (Figure 2). Physiological simulations showed E5P rates ≤ 2 Hz with low to intermediate E5P firing vector (FV) similarity. Dystonia simulations primarily occupied the upper-right quadrant of the scatterplot in Figure 2A, but displayed either high or low FV similarity which overlapped with the range of values displayed by the physiological simulations. Epileptiform simulations had intermediate average E5P rates due to high activity alternating with periods of quiescence caused by depolarization blockade. Across simulation types, higher E5P firing increased the excitatory drive to I5 neurons, causing increased I5 neuron firing (Table 8). Higher I5 and E5P neuron firing then caused higher E5P synchronization via recurrent E5P excitation and feedback inhibition (Figure 2B). Stronger E5P and I5 interactions then increased beta rhythm amplitude (Figure 2B), however with substantial variability. Peak oscillatory frequency was held relatively stable across simulations (Table 8). Physiological and epileptiform simulations had lower overall E5P synchrony and beta power compared to the dystonia simulations, which occupied the upper-right quadrant of Figure 2B.

E5P FV similarity showed temporal recurrences which further distinguished the three simulation types (Figure 2C). The physiological simulation showed intermediate self-similarity (0.17) due to sparse firing of different subsets of pyramidal cells at different times. In contrast, the dystonia simulation firing patterns showed strong self-similarity (0.56) and recurrence over time (recurring orange/red blobs in Figure 2C), indicating stereotyped dynamics. The example epileptiform simulation showed relatively weak self-similarity (0.16) due to its two distinct firing patterns: high E5P synchrony alternating with E5P silence produced by depolarization blockade. Epileptiform and dystonia simulations showed a brief period of high similarity when the epileptiform simulation showed strong oscillations during the initial period. There was weak similarity between epileptiform and physiological (0.12) and dystonia and physiological (0.22) simulations, indicating that both pathological dynamics were distinct from the physiological.

**Figure 3:**
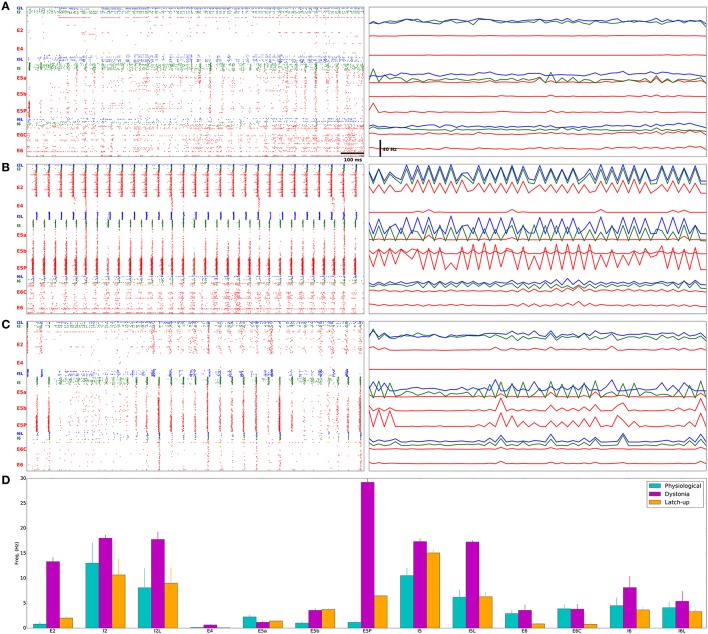
**Distinct firing patterns in physiological, dystonia, and epileptiform (epileptic) simulations**. **(A)** Physiological model has sparse, asynchronous E5P firing, relatively low I5 firing, and activated E5a/E5b populations. **(B)** Pathological model shows high-frequency, synchronous activity in E5P neurons, causing higher I5 firing, which suppresses E5a activity. **(C)** “Epileptiform” (epileptic) model shows high-frequency, synchronous activity with intermittent 200–300 ms gaps in firing of E neurons, caused by *depolarization blockade* (Na^+^ channel inactivation). **(A–C) Left** Dots represent individual neuron spike times (red: E cells, blue: LTS cells, green: FS cells). Cells arranged from layer 2/3 (top) to layer 6 (bottom). Scale-bar: 100 ms. **(A–C) Right** Population firing rates (25 ms bins) arranged vertically to roughly correspond to position on raster plot to the left. Scale-bar: 40 Hz (Same color code as raster; apparently flat lines indicate low variation in firing rate). **(D)** Population firing rates from simulations in **(A–C)** (Average ± standard error of the mean).

**Figure 4:**
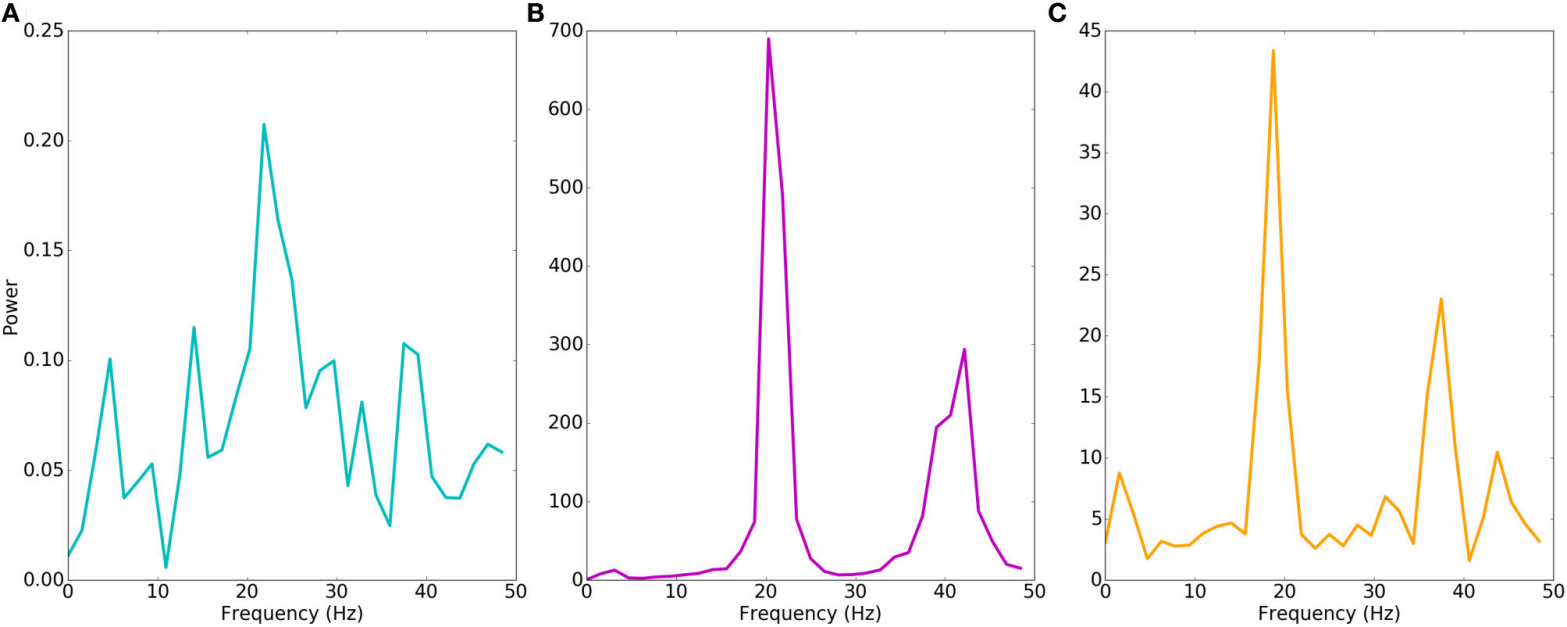
**Motor cortex models produce different beta oscillations**. Power spectrum of multiunit activity vectors of examples in Figure 3. Power (y-axis) in arbitrary units. **(A)** Physiological model shows weak beta (22 Hz) oscillations with power of < 0.1% of the pathological model. **(B)** Pathological model produces strong beta (20 Hz) oscillations with additional harmonic at 40 Hz. **(C)** Epileptiform model produces strong beta (19 Hz) oscillations with additional harmonic at 38 Hz.

E5P neurons in a representative physiological model fired sparsely with low synchrony (population-synchrony = 0.01; Figures 3A,D; Supplementary Figure 1 has all physiological rasters), with multiple downstream effects. Low excitatory drive from E5P to I5 and I5L neurons caused them to fire slowly. This low L5 inhibition allowed E5a neurons to fire quickly. The weak E5P and L5 interneuron interactions produced only weak beta rhythms which were confined to layer 5 (Figure 4A).

In a representative dystonia simulation, E5P neurons had sustained, synchronous, rapid firing (Figures 3B,D; Supplementary Figure 2 shows all dystonia simulation rasters). This promoted strong, continuous layer 5 interneuron activation. The L5 interneurons then suppressed E5a intratelencephalic neurons, which fired at reduced rates. In contrast, E5b firing increased with the faster E5P firing, due to excitation spreading in the network. The relatively high recurrent connectivity (18% density) and strong synaptic weights between E5P neurons allowed the E5P neurons to remain activated despite strong feedback inhibition. The strong feedback inhibition also activated the E5P HCN channels, which produced rebound excitation. The strong E5P activation coupled with the feedback inhibition also enabled E5P neurons to synchronize (population-synchrony = 0.83; vertical stripes in Figure 3B) at a strong beta rhythm (~20 Hz; Figure 4B). These synchronous beta rhythms also spread to other populations and layers (E2, I5, I5L, E5b, and E6).

Epileptiform simulation also displayed strong intermittent beta oscillations and strong synchrony (population-synchrony = 0.05; Figures 3C, 4C), but this activity alternated with lengthy periods (200–300 ms) where E neurons were not firing due to depolarization blockade. Even with these periods of depolarization blockade, most E neurons fired at higher average rate than in the physiological simulations (Figure 3D). Such increased synchrony with high excitatory cell activity is seen in epilepsy patients (Meisel et al., 2015). In contrast to the dystonia simulations, the synchronous periods of epileptiform oscillations were largely confined to layer 5 and did not spread to other layers.

### 3.3. Need for multitarget approach

**Figure 5:**
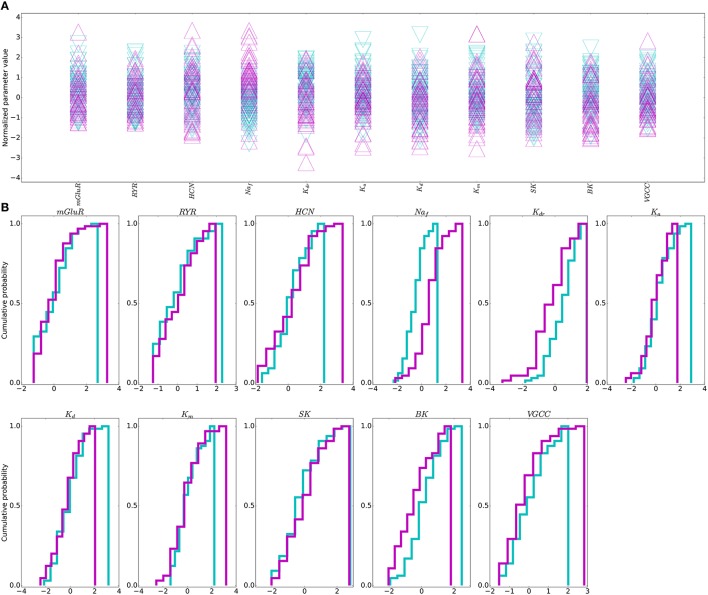
**Individual parameters do not distinguish physiological from dystonia activity**. **(A)** Dystonia (purple) vs. physiological (light blue) simulations. of simulations sorted by E5P firing rate (*N* = 65 for each group). **(B)** Cumulative probability distributions for each parameter in the dystonia (purple) and physiological (light blue) simulations. Parameter values normalized to a distribution with zero mean and unit variance (zero mean does not indicate zero density of a given ion channel/receptor). Simulations shown are obtained from bottom and top 2nd percentile based on dynamic measures.

No individual parameter determined physiological vs. dystonia-dynamical-condition in the network (Figure 5). Therefore, no single parameter adjustment would routinely provide an effective “treatment” that would reliably restore physiological activity in most pathological models. We therefore went on to explore whether multitarget manipulation would be able to find such treatment routes.

Although no single parameter could predict physiological vs. pathological dynamics, the outliers of certain individual parameters were predictive. At the pathological margin, simulations had parameters which are expected to produce high activity: high Na^+^ or Ca^2+^ channels promoting inward currents, high HCN channel densities providing high resting membrane potential (RMP), and low K^+^ channel densities again producing depolarization and reduced repolarization with spiking.

**Figure 6:**
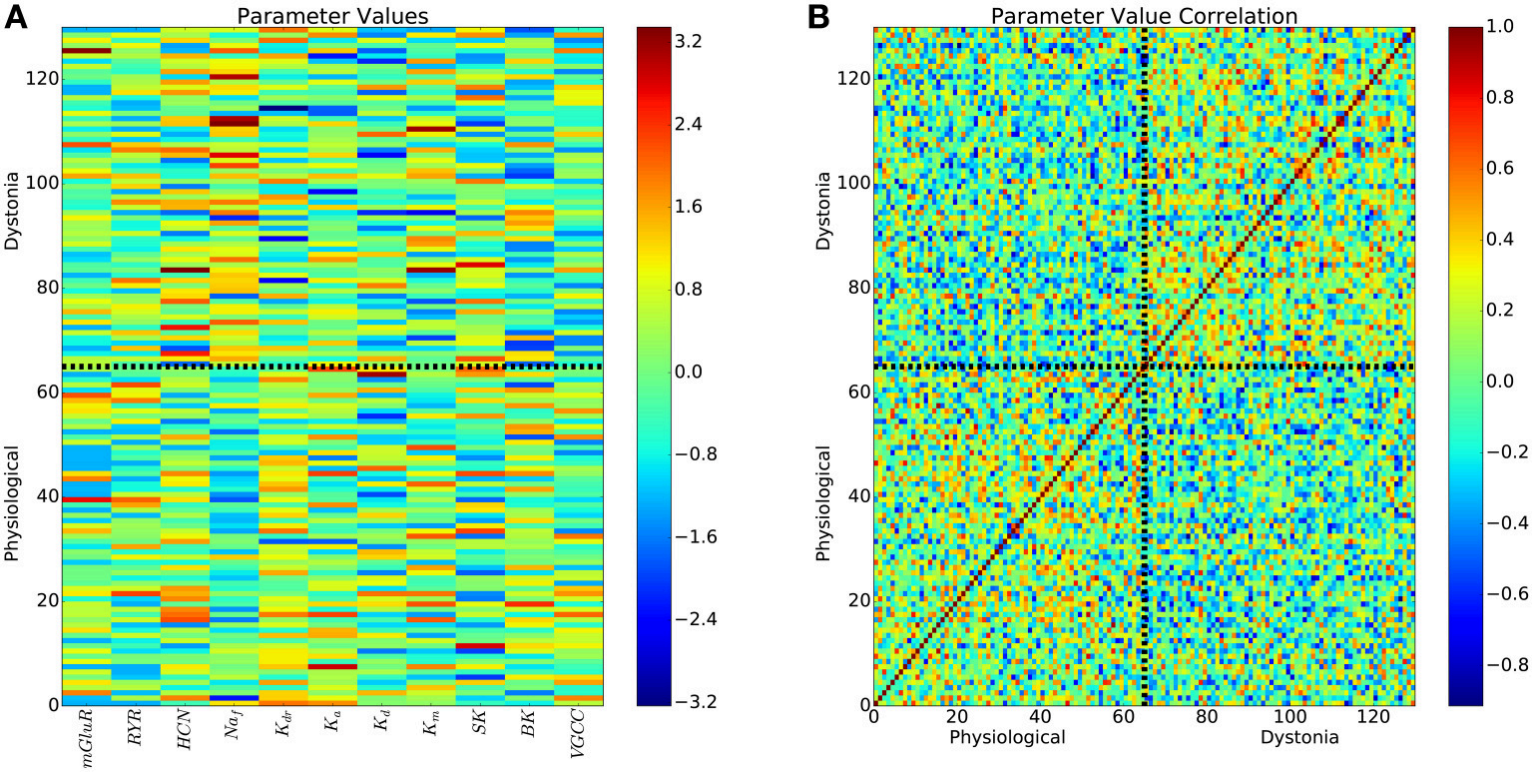
**All parameters of pathological and physiological simulations reveals weak intra-class clustering**. **(A)** 11-dimensional parameters for physiological and pathological simulations. Colorbar is normalized parameter values as in Figure 5. **(B)** Pearson correlations between all pairs of parameter vectors.

Further evidence for lack of predictability of dynamics based on parameters, comes from viewing the parameters in all 11 dimensions organized into 2 classes by dynamics. The parameter space showed substantial heterogeneity in the patterns producing pathology (Figure 6A), with weak intra-class clustering (Figure 6B). Correlation between parameter vectors of each simulation averaged 0.06 for physiological simulations, 0.07 for pathological simulations, with weak -0.05 anticorrelation between pathological and physiological simulations. The low correlations in both the physiological simulations (lower-left corner of Figure 6B) and the pathological simulations (upper-right corner of Figure 6B) demonstrate that there is widespread degeneracy in the parameter sets that produce either the physiological or pathological states. Some of this degeneracy is unsurprising: for example K^+^ channels with similar time courses of activation can substitute for one another to some extent. Other degeneracy is more complex and involves higher-order dynamic compensation.

### 3.4. High dimensional separation of physiological and pathological parameters

**Figure 7:**
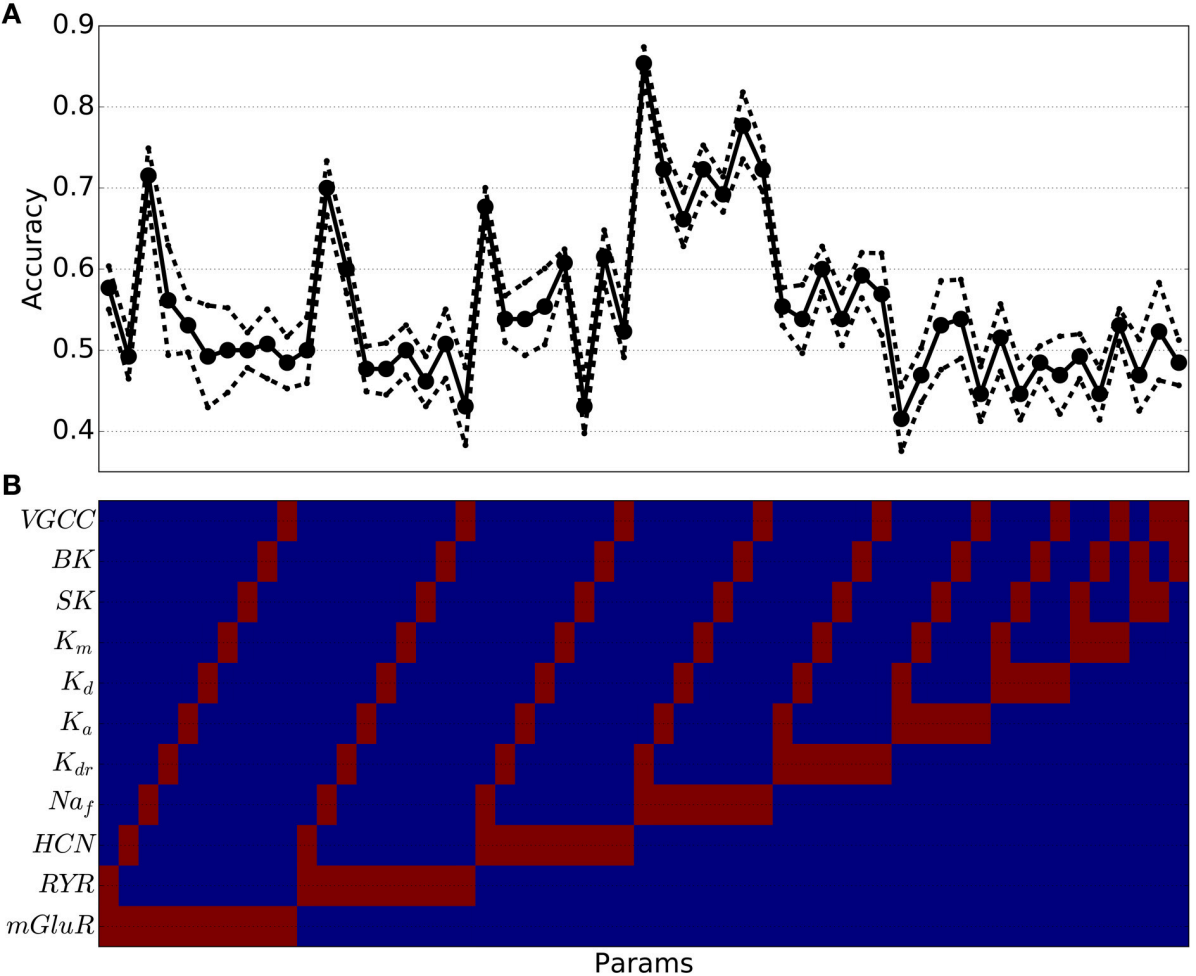
**Support vector machine classification accuracy of pathological vs. physiological simulations using two parameter values has high levels for certain parameter combinations (e.g., including Na_*f*_ channel density) but overall accuracy is often below chance (0.5)**. **(A)** Accuracy as a function of specific parameter combinations [indicated at same horizontal location in **(B)** (Red indicates parameter (param) was used for classification; blue indicates the parameter was not used)] (solid line: mean cross-validation accuracy (*n* = 10); dotted line: standard error of cross-validation accuracies).

Because of the difficulty of separating pathological from physiological with these high dimensional parameter sets, we used a SVM classification to create a separation (termed a maximum margin hyperplane) separating parameter sets producing physiological dynamics from parameter sets producing pathological dynamics. We started by training SVMs using only two parameters in combination (Figure 7). In order to test the efficiency of this separation, we separated out our target sets (physiological vs. pathological) into two subsets of each to serve as training and testing sets to evaluate the adequacy of the separation. By trying various random training and testing sets we got a mean and standard error for each case. Many two-parameter predictions were below chance (0.5) indicating that the SVM could not separate physiological from pathological based on that parameter pair. Two-parameter SVMs could accurately classify when the parameter pair included Na_*f*_ density—the strongest predictor of excitability. The best prediction came with high Na_*f*_ and low K_*dr*_. Logistic regression methods were also tried to perform this two-dimensional separation but did not perform as well as SVM.

**Figure 8:**
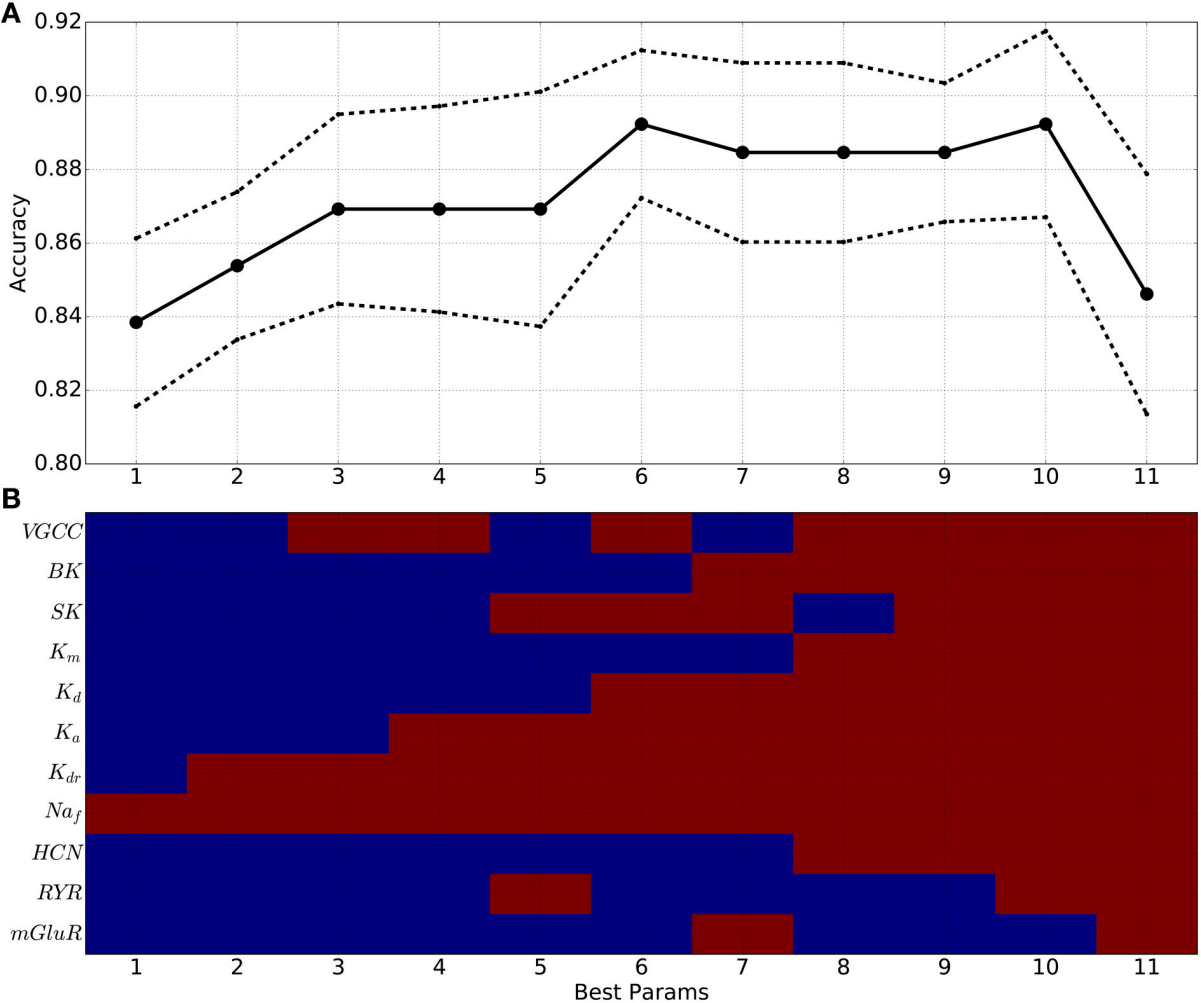
**SVM classification accuracy generally increases when using 1–10 parameters, indicating utility of multitarget pharmacy approach to treating dystonia**. **(A)** Best classification accuracy from all combinations of *x* parameters (solid line: mean cross-validation accuracy (*n* = 10); dotted line: standard error). **(B)** Best parameter (param) combinations (red: parameter used; blue: parameter not used). x-axis in **(A,B)** indicates number of parameters used.

Going beyond 2 parameters, SVM classification accuracy increased regularly with the number of parameters used (Figure 8), suggesting that a multi-target drug approach beyond two targets might produce greater effect. Moving to higher and higher dimensional spaces, we checked all possible parameter combinations at each dimensionality. In Figure 8, we report the parameter combination that was most predictive—e.g., at 6 dimensions we report just one of the 462 combinations of six from 11 parameters. Looking at the red blocks below, we can identify that the six dimensions that provide best prediction are Na_*f*_, four of the K^+^ channels, and VGCC. Predictability increases up through six parameters, then plateaus, and then falls off due to the extreme sparseness of data. This sparseness was due to the so-called curse of dimensionality: given a constant number of data points *n*, the density falls off *#bin*-fold with each increase in dimension, where *#bin* is the binning of the space in one dimension. Because of this, any high-dimensional method will tend to underestimate predictive strength given a limited amount of data (Bishop, 2006; Noble, 2006).

This multi-target SVM approach revealed the parameters that had the highest contribution to producing or preventing dystonia. Na_*f*_ density was the most predictive parameter across all numbers of parameters used (horizontal red stripe at top of Figure 8B), as had been also shown using 2 dimensions alone (Figure 7). Again confirming the 2-dimensional result, the next most predictive parameters was K_*dr*_. Following these came K_*a*_, K_*d*_, BK, SK, and VGCC densities which also significantly contributed to accurate predictions, due to their strong influence on E neuron excitability. mGLUR, RYR, and K_*m*_ densities showed lesser contributions.

**Figure 9:**
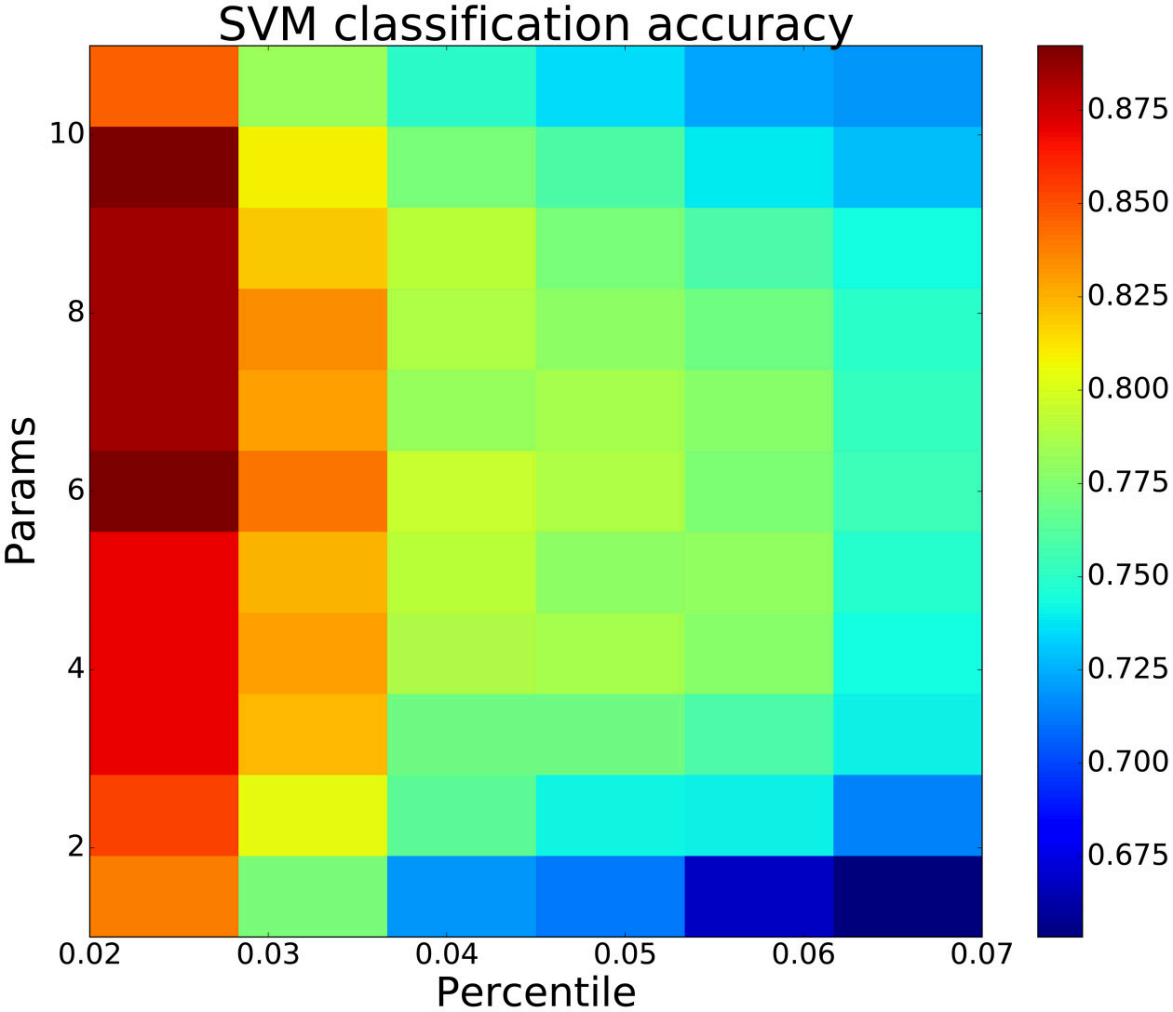
**SVM classification accuracy increases with more parameters then decreases due to “curse of dimensionality”—sparseness of parameter vectors relative to dimension**. Best classification accuracy from all combinations of *y* parameters (params) using bottom/top SPI firing rate percentiles on x-axis.

Increasing the percentile cutoffs for categorizing physiological from pathological simulations from the 2nd percentile to 7th percentile decreased prediction accuracy but still demonstrated the value of multitarget changes (Figure 9). The left column shows the same result as Figure 8: accuracy increased (colormap) as one goes from fewer to more parameters (bottom to top). By including more exemplars on both the physiological and pathological sides, we moved away from the best exemplars and obtained less distinction between the two parameter sets. However, at all percentiles, there was an initial increase in classification accuracy with continued increase up to or beyond 3 parameters. This increase then declined as the number of parameters increased further due to the aforementioned sparseness at high dimensionality.

## 4. Discussion

We developed a multiscale model of primary motor cortex to explore multitarget pharmacological therapies for dystonia. We searched parameter space—channel and receptor densities—to create a set of models to contrast dystonia dynamics with physiological dynamics. Dystonia simulations displayed high excitability and synchrony in layer 5 corticospinal neurons (E5P), and strong beta oscillations which spread between cortical layers (Figures 3B, 4B). Dystonia simulations could be distinguished from epileptiform simulations primarily by the presence of periods of latch-up with depolarization blockade in the epileptiform simulations. Physiological simulations had low excitability, asynchronous firing, and weak beta oscillations (Figures 3C, 4C). Attempts to use high-dimensional visualization techniques to find potential therapeutic directions in the parameter space were limited by the solution degeneracy in the 11-dimensional parameter space with scattered parameter vectors with low correlation (Figure 6). We therefore turned to a SVM classification to identify hyperplanes in high-dimensional space that would separate the two populations. As expected, the major spike generating channels, Na_*f*_ and K_*dr*_ were the primary determinants of excitability, followed by additional potassium channels and calcium channels. We did not assess pharmacological effects on synapses, which would be useful to do in the future.

### 4.1. Biological degeneracy and personalized therapy

Degeneracy of mechanism is a major theme in biology (Edelman and Gally, 2001), meaning that there are many different ways that a biological system can produce a particular shape in the case of an immunoglobulin, or a particular dynamics in the case of a neural system. Such degeneracy has been shown directly in the stomatogastric ganglion of lobster, where a particular cell type produces its stereotyped dynamics using many different combinations of ion channel densities (Golowasch et al., 2002). Associated with this degeneracy is *failure of averaging*—averaging across parameter sets that produce the dynamics gives a set of parameter values that do not produce the same dynamics.

In the context of brain physiology, this means that we can expect that individuals differ in the details of how their motor cortex produces oscillations and contributes to movement. Similarly, we can expect that individuals differ in the details of their pathology. From a pharmacological perspective this argues that we may see greater benefit from personalized medicine—identifying the high-dimensional complex of pathological parameters in a particular patient in order to treat them with their own individualized cocktail of multitarget drugs to produce a dynamics that falls somewhere in the physiological regime. To this might also be added complementary individualized, perhaps multi-locus, brain stimulation (Kerr et al., 2012; Song et al., 2013; Chadderdon et al., 2014; Hiscott, 2014; Nelson and Tepe, 2014; Dura-Bernal et al., 2016). Such a personalized approach would require much more intensive, and more costly, diagnostic procedures of a type that is currently only used by epilepsy surgery centers, which typically require invasive methods to perform their diagnostic tests.

Due to the degeneracy, parameter averaging failed in our dataset—using the average of all parameters sets that produce pathological simulations does not give a pathological simulation. However, the ability of the SVM method to separate pathological from physiological populations in high dimensional parameter space does suggest that there may be some benefit to pushing all patients in that direction through a multitarget pharamacological cocktail. In future studies, we plan to test this explicitly in the simulations in order to determine what percentage improve, what percentage worsen and what percentage remain essentially unchanged with such an average treatment. This assessment will require a larger number of simulated patients than we have thus far accumulated.

### 4.2. Multilocus, multitarget, multiscale approaches for treating dystonia

In general, single target pharmacology has not been effective in dystonia (Fahn, 1987). As in other complex diseases, many of the treatments for dystonia have highly variable effectiveness and must be used at high doses that produce side-effects (Jankovic, 2006). For these reasons, botulinum toxin, targeting the final endpoint —the muscle movement—is commonly used as a treatment (Jankovic, 2006; Sanger et al., 2007; Bragg and Sharma, 2014). Deep-brain stimulation, an invasive procedure, is also used to partially restore normal brain dynamics (Tarsy, 2007; Johnson et al., 2008; Air et al., 2011; Bhanpuri et al., 2014).

Multilocus, multitarget approaches may be particularly useful in movement disorders because movement produces coordination by utilizing coordination among multiple brain areas including the basal ganglia, thalamus, cerebellum, sensory, and motor cortices (Neychev et al., 2008; Crowell et al., 2012; Delnooz and van de Warrenburg, 2012). Pathology within any one region, or disturbances in communication between any of the regions can potentially lead to disorders. To begin to address these multiple challenges, we focused our computer modeling here on a multiscale model of motor cortex and multitarget pharmacology based in this one area. In the future, this model will be expanded to encompass more areas and will include synaptic receptor targets in each area.

## Author contributions

All authors listed, have made substantial, direct and intellectual contribution to the work, and approved it for publication.

### Conflict of interest statement

The authors declare that the research was conducted in the absence of any commercial or financial relationships that could be construed as a potential conflict of interest.
