# Supplementary figures and images for: Multitarget Multiscale Simulation for Pharmacological Treatment of Dystonia in Motor Cortex
Source: Front Pharmacol. 2016 Jun 14;7:157. doi: 10.3389/fphar.2016.00157 (PMC4906029; doi:10.3389/fphar.2016.00157)

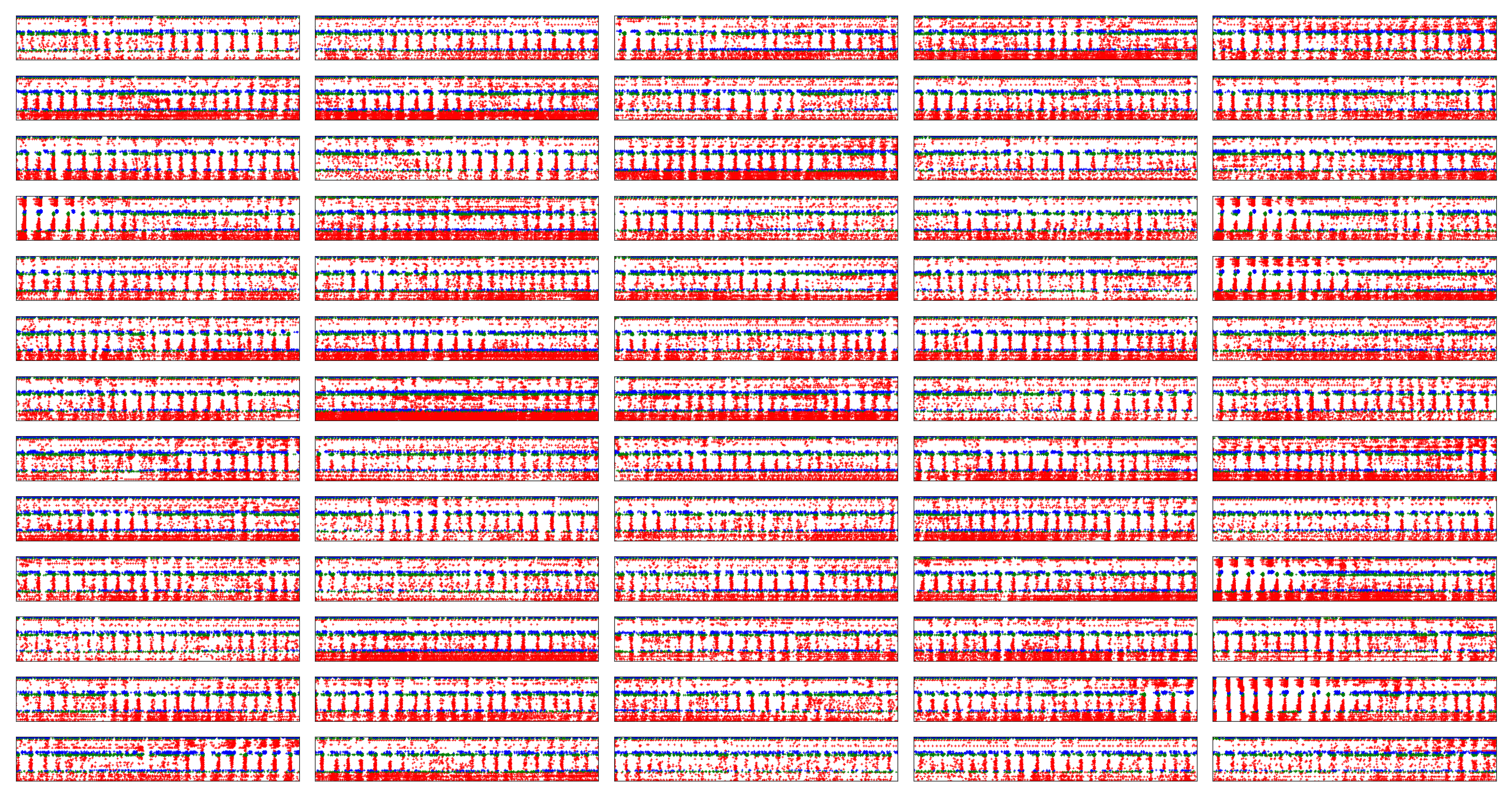

Supplement: Supplementary file 1 [file Image1.PNG]

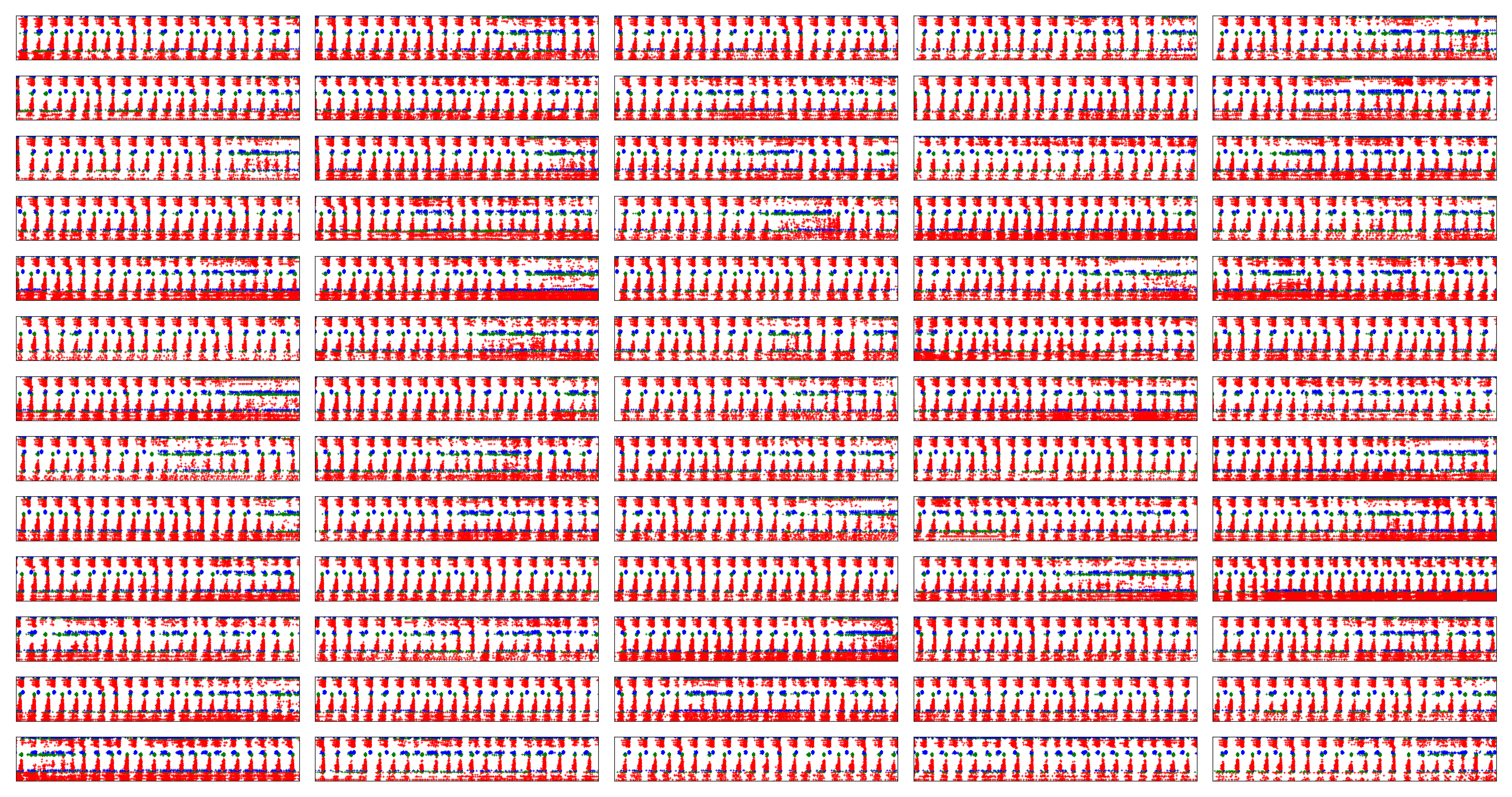

Supplement: Supplementary file 2 [file Image2.PNG]
